# Xeno-free culture condition for human bone marrow and umbilical cord matrix-derived mesenchymal stem/stromal cells using human umbilical cord blood serum

**Published:** 2016-09

**Authors:** Azadeh Esmaeli, Mojgan Moshrefi, Ali Shamsara, Seyed Hasan Eftekhar-vaghefi, Seyed Noureddin Nematollahi-mahani

**Affiliations:** 1 *Physiology Research Center, Institute of neuropharmacology, Kerman University of Medical Sciences, Kerman, Iran. *; 2 *Kerman Student Research Center, Kerman University of Medical Sciences, Kerman, Iran. *; 3 *Research and Clinical Center of Infertility, Shahid Sadoughi University of Medical Sciences, Yazd, Iran.*; 4 *Neuroscience Research Center, Institute of Neuropharmacology, Kerman University of Medical Sciences, Kerman, Iran. *; 5 *Department of Anatomy, Afzalipour School of Medicine, Kerman University of Medical Sciences, Kerman, Iran.*

**Keywords:** *Bone marrow*, *Cell proliferation*, *Cord blood*, *Mesenchymal stem/stromal cells*, *Umbilical cord*, *Wharton jelly*

## Abstract

**Background:**

Fetal bovine serum (FBS) is widely used in cell culture laboratories, risk of zoonotic infections and allergic side effects create obstacles for its use in clinical trials. Therefore, an alternative supplement with proper inherent growth-promoting activities is demanded.

**Objective:**

To find FBS substitute, we tested human umbilical cord blood serum (hUCS) for proliferation of human umbilical cord matrix derived mesenchymal stem cells (hUC-MSCs) and human bone marrow-derived mesenchymal cells (hBM-MSCs).

**Materials and Methods:**

Umbilical cord blood of healthy neonates, delivered by Caesarian section, was collected and the serum was separated. hUC-MSCs and hBM-MSCs were isolated and characterized by assessment of cell surface antigens by flow cytometry, alkaline phosphatase activity and osteogenic/adipogenic differentiation potential. The cells were then cultured in Iscove's Modified Dulbecco's Medium (IMDM) by conventional methods in three preparations: 1- with hUCS, 2- with FBS, and 3- without serum supplements. Cell proliferation was measured using WST-1 assay, and cell viability was assessed by trypan blue staining.

**Results:**

The cells cultured in hUCS and FBS exhibited similar morphology and mesenchymal stem cells properties. WST-1 proliferation assay data showed no significant difference between the proliferation rate of either cells following hUCS and FBS supplementation. Trypan blue exclusion dye test also revealed no significant difference for viability between hUCS and FBS groups. A significant difference was detected between the proliferation rate of stem cells cultured in serum-supplemented medium compared with serum-free medium.

**Conclusion:**

Our results indicate that human umbilical cord serum can effectively support proliferation of hBM-MSCS and hUC-MSCs in vitro and can be used as an appropriate substitute for FBS, especially in clinical studies.

## Introduction

Human stem cells (HSCs) are characterized by their potency for self-renewal and their capacity to differentiate into various cells types (1, 2). Up to now, the most common sources of HSCs for transplantation were bone marrow and peripheral blood cells. But emerging evidences have recommended human umbilical cord blood hematopoietic stem cells (hUC-HSCs) and human umbilical cord matrix-derived mesenchymal stem cells (hUC-MSCs) for allogenic transplantation (2-4). However, collection of bone marrow is an invasive and painful procedure. In addition, the numbers of bone marrow mesenchymal stem cells decreases significantly with age and infirmity. Furthermore, there is a high risk of viral contamination during mesenchymal stem cells (MSCs) collection. All these factors limit the use of bone marrow, thus, there is a need for cells with higher proliferative potency and differentiation capacity with lower risk of viral contaminations (2, 5, 6). 

Compared with bone marrow stem cells, hUC-MSCs and hUCB cells are more suitable sources of stem cells with minimum, if any, immune response following xenotransplantation, as well as a higher proliferation capacity and possible in vitro and in vivo differentiation into various cell types, including muscle cells, neurons, cardiomyocytes, etc. (7-9). 

Serum, in general, is one of the crucial elements in animal cell culture to support survival and proliferation of the cells. Fetal calf serum (FCS) and fetal bovine serum (FBS) are the most popular types of serum which are using routinely in animal cell culture laboratories (10). Serum-free medium and 5% FBS supplementation of medium has resulted in 88% and 12% proliferation rate of cultured cells, respectively. Also, by increasing the amount of FBS from 10-20%, proliferation rate of cultured cells has increased at later passages (11). 

In most culture systems, FBS/FCS is an essential component for cells growth and maintenance. It contains low and high molecular weight biomolecules and a variety of factors that promote or inhibit growth. Low toxicity and growth-promoting properties of FBS/FCS make it a common supplement for in vitro culture of most mammalian cells. However, animal sera are a potential source of microbial and viral contaminants, particularly mycoplasma, bovine viruses, and other pathogens (21). Suppliers use a variety of techniques, including irradiation, filtration, and/or heat-inactivation to reduce any microbial contamination but the use of FBS/FCS is not recommended in human cell transplantation (12-15). Moreover, application of FBS/FCS in clinical procedures may lead to local inflammation, stimulation of immune responses against xenoproteins, and tissue rejection due to xenogenic immune reactions. The disadvantages of FBS/FCS cast doubt on its biosafety in cell therapy procedures (21).

Several alternatives for FBS/FCS have yet been suggested. These include supplementation of culture media with growth factors such as IL-6, IL-11, IL-12, thrombopoietin, leukemia inhibitory factor and granulocyte colony-stimulating factor which are not cost effective (16, 17). Some human autologous serum derivatives such as platelet lysate, a safer and less expensive substitute, were successfully used for the culture of MSCs and corneal epithelial cells (18-22). Peripheral blood plasma was also used for culture of rat stem cells, but the results were not reasonable (23). Another alternative could be human umbilical cord serum (hUCS), but studies on hUCS are limited (14).

To the best of our knowledge, there is no published report in the literature to describe the effects of hUCS on the proliferation of hUC-MSCs cells. We designed this study to investigate the potential of hUCS, in comparison with FBS, as a supplement for the proliferation of hUC-MSCs and human BM-MSCs. To assess this hypothesis, we cultured two different types of mesenchymal stem cells in serum-free, FBS-supplemented and hUCS-supplemented conditions and the results were evaluated with a proliferation assay kit and trypan blue staining.

## Materials and methods

In the present experimental study all the chemicals were purchased from Sigma (Sigma Aldrich Chemical Company, St Louis, MO, USA) unless stated otherwise. The Ethics Committee at Kerman University of Medical Sciences approved this study. The written consent was obtained from all participants.

**Human umbilical cord serum preparation**

To prepare the hUCS, hUCB was taken from neonates whose mothers had no medical history of serious disease. Cord blood samples were centrifuged at 450 gr for 10 min and supernatant was then collected and stored at -20^o^C. Samples were examined for mycoplasma contamination.

**Culture of human BM-MSCs**

Bone marrow samples from healthy individuals were obtained after fully informed consent. 20 ml bone marrow was aspirated from the iliac crest into sterile heparinized tube and immediately transferred to the laboratory, mixed with an equal volume of phosphate buffered saline (PBS) and centrifuged at 350 gr for 10 min. The nucleated cells at the interference between the red cells and PBS were removed carefully and transferred into a 75 cm^2^ flask. 15 ml complete culture medium was added to the flask. 

The complete culture medium consisted of Iscove's Modified Dulbecco's Medium (IMDM) supplemented with 10% FBS (Gibco, Grand Island, NY, USA), 100 IU/ml penicillin, 60 µg/ml streptomycin sulfate, and 50 µg/ml amphotericin B. Culture flasks were incubated at 37^o^C in the humidified air with 5% CO_2_. After 24 hr, the floating cells were removed, while the attached cells were washed three times with pre-warmed PBS. The flasks were then filled with 15 ml of complete medium supplemented with 1:100 insulin-transferrin-selenium solutions and incubated at the same conditions. 

The medium was refreshed every 4-6 days according to the rate of cell propagation. After reaching >90% confluence, the cells were detached from the substratum by 3 ml 0.25 g/l trypsin and 0.2 g/l EDTA. 

**Culture of hUC-MSCs**

hUC-MSCs were harvested using a previously reported method (8, 24). Briefly, umbilical cords of neonates that were delivered by Cesarean section were transported in PBS to laboratory. Umbilical cord vessels and amniotic membrane were separated under sterile conditions. The remaining matrix was divided into small pieces (>1 mm) and loaded onto the bottom of 6 cm petri dishes. 

1 ml complete medium was added to each petri dish and the dishes were incubated at 37^o^C humid atmosphere with 5% CO_2_ in the air for 24 hr. 7 ml of complete medium was then added to each petri dish and after 1 wk, matrix pieces were removed. We used cells at passage 2-7 in our experiments. The mitotic index was calculated as the proportion of the number of cells undergoing mitosis to total number of cells present in the microscopic field.

**hUC-MSCs characterization**

hUC-MSCs were characterized according to their capacity to form colonies, alkaline phosphatase activity and the expression of cell surface markers. We used a frozen batch of hUC-MSCs with confirmed cell surface markers for the experiments (3, 24). Cell surface markers of BM-MSCs were also characterized in a previously published study (25).

**Adipogenic and osteogenic differentiation**

**Osteogenic assay**

hBM-MSCs and hUC-MSCs were seeded at 2×10^4^ cells/cm^2^ in culture dishes. After 72 hr, the medium was replaced by osteogenic medium; DMEM-F12 with 10% FBS, 50 µg/ml gentamycin, 10 µM β-glycerophosphate, 10^-7^ M dexamethasone and 0.2 mM ascorbic acid. The medium was refreshed every 3-4 days and on day 21, the cells were processed for differentiation analysis by Alizarine red staining.

**Adipogenic assay**

hBM-MSCs and hUC-MSCs were seeded at 2×10^4^ cells/cm^2^ in culture dishes. After 24 hr, the medium was replaced by Adipogenic medium; DMEM-F12 with 10% FBS, 0.01 µM dexamethasone, 0.5 mM IBMX (3-isobutyl-1-methyl xanthine) and 60 µM indomethacin. Adipogenic medium was refreshed every 3-4 days and on day 14, the cells were processed for differentiation analysis by oil red staining.

**WST-1 cell proliferation assay**

The WST-1 assay was designed to evaluate the rate of cell proliferation, growth and viability. We cultured 1×10^4^ viable cells in each of the 96-well plates with a final volume of 100 μl IMDM medium supplemented with 10% FBS, hUCS or no supplement as control. Plates were incubated at 37^o^C and 5% CO_2_ for 48 hr. At the end of the incubation period, 10 μl WST-1 cell proliferation reagent was added to each well and the plates were incubated in humid atmosphere at 37^o^C for 1 hr. 

Plates were vortexed slowly and absorbance of the samples was measured using an ELISA reader instrument (BioTek Elx800, USA) with 450 nm wavelength and a reference value of 630 nm (26). Experiments were replicated at least 4 times in quadruplicate. To nullify the effects of culture media absorbance during WST-1 assay, we measured the absorbance of 100 µl IMDM with 10% FBS or hUCS and subtracted it from the absorbance of cell-containing assay.

**Trypan blue cell viability assay**

The cells cultured for 48 hrs in different treatment groups at standard culture condition were disaggregated using trypsin/EDTA and total cell count was determined using a haemocytometer chamber after staining the cells with 0.4% trypan blue. An equal volume of cell suspension was mixed with 0.4% trypan blue and the mixture was loaded onto an improved Neubauer haemacytometer. Unstained cells were considered viable and those colored blue were considered dead. 

**Experimental design**

The proliferation of hBM-MSCs and hUC-MSC cells was compared in different groups as follows. 1) hUCS/BM-MSCs group in which BM-MSCs were treated with IMDM medium supplemented with 10% hUCS, 2) FBS/BM-MSCs group in which BM-MSCs were treated with IMDM supplemented with 10% FBS, 3) IMDM/BM-MSCs in which BM-MSCs were treated with IMDM without serum supplement as control, 4) hUCS/hUC-MSC group in which hUC-MSCs were treated with IMDM supplemented with 10% hUCS, 5) FBS/hUC-MSC in which hUC-MSCs were treated with IMDM supplemented with 10% FBS and 6) IMDM/hUC-MSC in which hUC-MSCs were treated with IMDM without serum supplement as control. 

**Statistical analysis**

Data were expressed as mean±SEM. Normality of the data was assessed by Shapiro-wilk test. One way ANOVA was used for comparison of the data in the different groups followed by Tukey post-hoc test. The proportion of viable cells in the different groups was statistically analyzed by ^2^ test. A statistical difference of p<0.05 was considered significant.

## Results

**Cells isolation and alkaline phosphatase activity**

**Figure 1:**
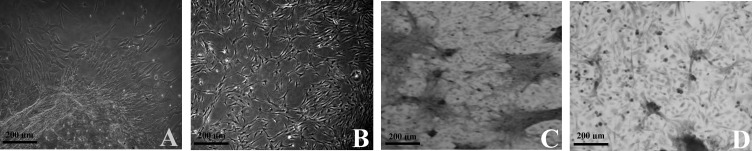
Cells morphology and alkaline phosphatase activity in cultured cells. The isolated cells derived from Wharton^'^ s jelly, exhibited fibroblastic like form (A). The BM-MSCs also appeared spindle shape and fibroblast-like in the culture (B). Positive alkaline phosphatase activity in hUC-MSCs (C) and BM-MSCs (D) was noted

The hUC-MSC cells isolated from Wharton's jelly appeared with a fibroblast like shape (Figure 1A). Also, the bone marrow cells demonstrated fibroblastic like morphology and spindle shape (Figure 1B). Both groups of the cells were positive for alkaline phosphatase activity as determined by R87 kit (Figure 1C, 1D).

**Cell propagation and colony formation**

**Figure 2:**
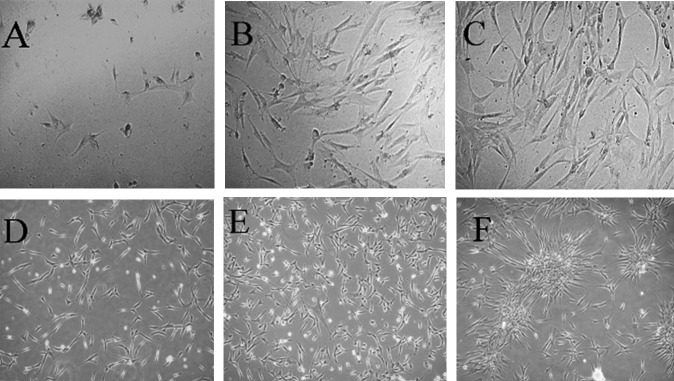
Morphology and colony formation properties of hBM-MSCs and hUC-MSC.

BM-MSCs propagated slowly in culture with a heterogeneous morphology of different cell types. After three days of culture, fusiform cells with abundant cytoplasmic processes as well as few round small shiny cells were appeared. BM-MSCs that were cultured in serum-free medium (Figure 2A), in 10% FBS (Figure 2B) or in 10% hUCS (Figure 2C), showed nearly similar morphology but the rate of proliferation was much slower in serum-free culture. Also, more fusiform cells were detectable in serum-free group.

In addition, mitotic index was calculated as the proportion of cells undergoing mitosis to the total number of the cells. Mitotic index was not high in BM-MSCs. However, by increasing the cell number, mitotic index increased. These cells scarcely showed tendency to make colonies in both hUCS and FBS supplemented media. A confluence of >80% was reached after 2-3 wks of primary culture. hUC-MSCs propagated more rapidly compared with BM-MSCs, and a higher mitotic index was detected compared to BM-MSCs. 

These cells also showed a mesenchymal appearance in serum-free culture medium (Figure 2D), 10% FBS- supplemented medium (Figure 2E) and 10% hUCS- supplemented medium (Figure 2F). These cells formed colonies following confluence of ≥80% in the presence of hUCS (Figure 2F).

**Cell surface markers**

hUC-MSCs and hBM-MSCs had expressed mesenchymal stem cell markers 

**Adipogenic and osteogenic differentiation**

**Adipogenic differentiation**

**Figure 3:**
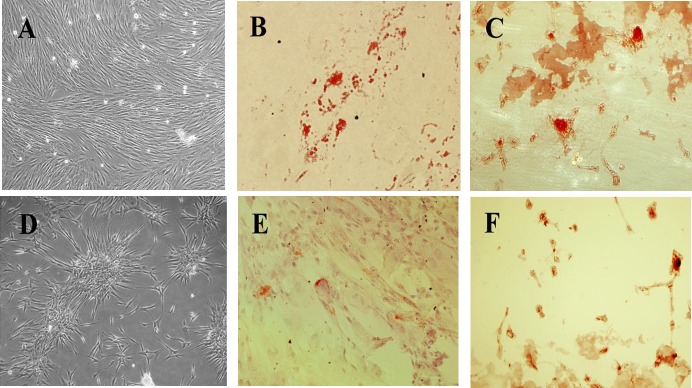
Adipogenic and Osteogenic differentiation of hBM-MSCs and hUC-MSCs.

After 14 days of incubation in the adipogenic medium, hBM-MSCs and hUC-MSCs showed fibroblastic morphology with more rounded contours and intracellular shiny lipid droplets, as seen in phase contrast microscopy. These lipid droplets stained red following Oil Red O staining (Figure 3B, 3E).

**Osteogenic differentiation**

To induce osteogenic differentiation, hBM-MSCs and hUC-MSCs were cultured in osteogenic medium for three weeks, after which the cells showed a more polygonal shape with extensive cytoplasmic processes. Alizarin red staining demonstrated that calcium phosphate mineralization was formed as a response to the inductive signals (Figure 3C, 3F). Mineralization was not observed in the control slides (phase contrast view), (Figure 3A, 3D).

**Effect of FBS and hUCS on proliferation of hBM-MSCs**

**Figure 4:**
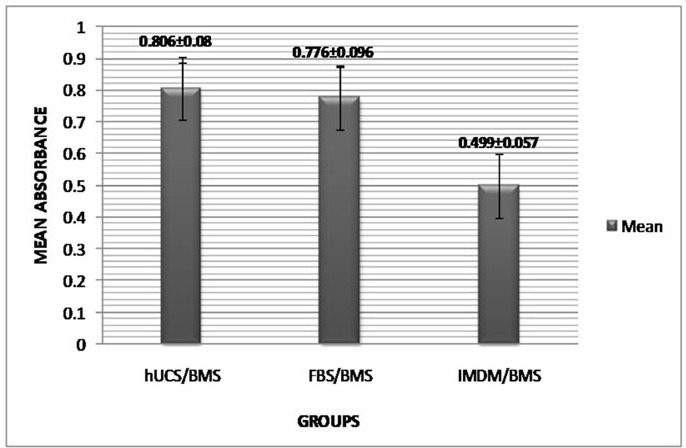
Effects of hUCS and FBS on proliferation of hBM-MSCs. Proliferation assay by WST-1 reagent showed similar proliferation rate in hUCS/hBM-MSC and FBS/hBM-MSC groups. A significant difference (P<001) was observed between IMDM/hBM-MSC and serum-supplemented groups

hBM-MSCs were cultured in the presence of FBS and hUCS or serum-free medium for 48 hr after which WST-1 assay was carried out. Results are presented in Figure 4. Mean absorbance was 0.777±0.512 for FBS/hBM-MSCs and 0.806±0.48 for hUCS/hBM-MSCs. We found no statistically significant difference between these groups (p=1). The cell proliferation was greatly dependent on serum supplementation. 

A significant decrease in cell proliferation rate was detected when the absorbance in serum-free medium was compared with the absorbance in serum-supplemented media (Figure 4).

**Effect of FBS and hUCs on cell viability of hBM-MSCs**

**Figure 5:**
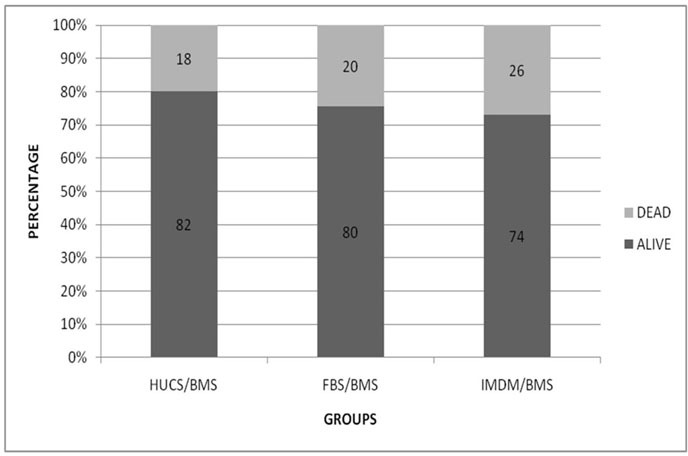
Tripan blue viability assay in hBM-MSC. Supplementation of culture medium with hUCS resulted in higher number of viable cells but it was not statistically different in comparison with FBS supplemented medium and serum-free medium

Trypan blue assay results with hBM-MSC are demonstrated in Figure 5. The proportion of viable cells was 80% in FBS/hBM-MSC, and 82% in hUCS/hBM-MSC. The difference between the studied groups was not statistically significant but was significantly (p<0.001) higher than serum-free medium (Figure 5). 

**Effect of FBS and hUCS on proliferation of hUC-MSCs**

**Figure 6:**
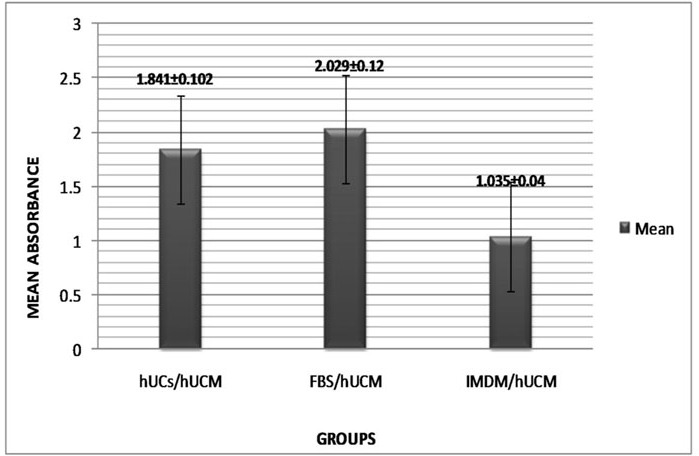
Effects of hUCS and FBS on proliferation of hUC-MSCs. Proliferation assay by WST-1 reagent showed similar proliferation rate in hUCS/hUC-MSC and FBS/hUC-MSC groups. A significant difference (P<001) was observed between IMDM/hUC-MSC and serum-supplemented groups

hUC-MSCs were also cultured in the presence of FBS, hUCS and serum-free IMDM medium for 48 hr and WST-1 assay was carried out. The mean absorbance of different samples is presented in figure 6. Mean absorbance readings was 2.029±0.509 for FBS/hUC-MSC and 1.841±0.422 for hUCS/hUC-MSC. But a significant difference (p<0.05) was observed between serum-supplemented (hUCS/hUC-MSC; FBS/hUC-MSC) and serum-free (IMDM/hUC-MSC) groups. 

**Effect of FBS and hUCS on viability of hUC-MSCs**

**Figure 7:**
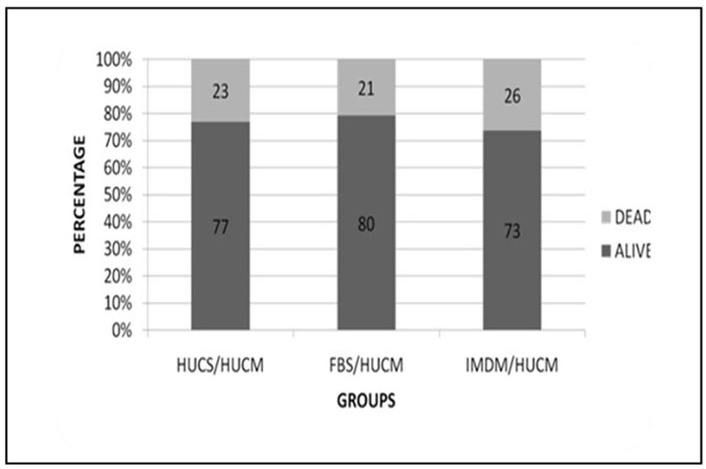
Tripan blue viability assay in hBM-MSCs. More viable cells were detected in FBS supplemented (FBS/hUC-MSC) group than other groups. However, it was not statistically different when compared with hUCS supplemented (hUCS/hUC-MSC) and serum-free (IMDM/hUC-MSC) groups

The results of trypan blue assay with hUC-MSCs are demonstrated in figure 7. Cell viability was 80% in FBS/hUC-MSC and 77% in hUCS/hUC-MSC. Cell viability was nearly identical in the serum supplemented groups, and there was no significant difference between serum supplemented groups and serum-free group (IMDM/hUC-MSC).

Morphology of the hBM-MSCs cultured either in serum-free IMDM (A), in 10% FBS (B) or in 10% umbilical cord blood serum (C), was nearly similar. Morphology of the hUC-MSCs cultured either in serum-free IMDM (D), in 10% FBS (F) or in 10% umbilical cord blood serum (G) was also nearly the same.

Phase contrast picture of hBM-MSCs (A) Oil red staining following adipogenic differentiation (B) and Alizarin red staining following osteogenic differentiation (C). Phase contrast picture of hUC-MSCs (D) Oil red staining following adipogenic differentiation (E) and Alizarin red staining following osteogenic differentiation (F).

## Discussion

In this study hBM-MSCs and hUC-MSCs were cultured in media supplemented with either hUCS or FBS. hUC-MSCs have recently been introduced as a promising source of stem cells in order to use in regenerative medicine (2, 8, 27). Compared with other different types of MSC including BM-MSCs, hUC-MSCs are not well described and the proliferative capacity of various natural sera has not been evaluated for these cells. Till now, hUCS efficacy has been examined for the culture of BM-MSCs from different species (28). Also, it was successfully used for proliferation of human limbal epithelial cells, neural precursor cells, skeletal mesenchymal cells, synovial mesenchymal stem cells, adipose stem cells and adipogenic differentiation, but no study has addressed umbilical cord serum efficacy for hUC-MSCs (29-33). 

According to our results, cell growth intensity and proliferation capacity was highly dependent on serum supplementations. The proliferation rate of hBM-MSCs and hUC-MSCs was considerably lower in the serum-free groups than theserum-supplemented groups. These data support previous findings regarding the vital role of serum for cell survival and proliferation in vitro (10, 18, 20, 35). FBS is a type of blood serum which contains most of the plasma materials including glucose, hormons, carbon dioxide, electrolytes (mainly Na+, Ca^2+^, Mg^2+^, HCO^3-^, Cl^-^) and proteins like globulin, albumin and fibrinogen except clotting factors (36). FBS is important for induction of some adhesive molecules expression (37).

But, zoonose contaminants, having animal source and immuno stimulating property are FBS controversies which makes it unsafe in human clinical trials, but in contrast, might suggest sera with human origin safer for clinical managements (12, 14). In addition, FBS contains unknown components that may carry some materials with advers effects into the cells (38). 

Besides, FBS changes the gene expression pattern in some group of cells (4). In this regard, FBS increases some gene expression like mesodermal differentiation-associated genes and decreases thrombin-activated platelet plasma (37). Investigations has revealed that FBS is unsuitable for pancreatic differentiation (34). Human serum exhibits higher capacity for adipogenic differentiation of adipose derived cells (33). But in osteogenic and chondrogenic differentiation of human synovium-derived mesenchymal stem cells, no significant differences between human serum and FBS was reported. Therefore when cell differentiation is assessed, FBS omission is preferable (32). The growth and proliferation of hBM-MSCs was slightly higher in media supplemented with hUCS determined by WST-1 assay. WST-1 assay is usualy used to determine in vitro cell proliferation and viability. 

In proliferating cells, tetrazolium salts are reduced by mitochondrial enzymes present in living cells. The higher absorption (higher OD) is correlated with greater number of viable and active cells in the sample (39). The trypan blue assay results were also in favor of this observation. But, for hUC-MSCs, the result was different, in which the proliferation rate was not significantly lower in the hUCS-supplemented medium compared with FBS. However, in all cases the inter-group differences were not statistically different. 

It has been shown that swine MSCs did not proliferate differently in the presence of hUCS or FBS, while human MSCs showed significant differences (29). More investigations are required to generalize our results to the other cell types. However, due to the fear of zootonic contaminations it can be concluded that animal sera are more appropriate for animals cell cultures and human sera are more suitable for human cell cultures (28). 

Although we did not find any significant difference between hUCS and FBS in terms of cell proliferation, other issues must be considered, and might suggest hUCS as a more appropriate substitute for FBS. Among human sera, fetal sera support cell proliferation better than adult sera because of higher concentrations of growth and differentiation factors and lower concentrations of gamma-globulins (14). We did not measure the gamma-globulin titer in hUCS, neither did we inactivate hUCS; an usual process that is commonly applied for any type of sera, but the propagation rate of hBM-MSCS and hUC-MSCs in the presence of hUCS was as high as FBS treatment. 

Umbilical cord matrix-derived cells originate early (around day 12 of gestation) in embryogenesis from extra-embryonic mesoderm. Due to their embryonic nature, hUC-MSCs are less likely to induce immune responses or produce tumors in the host. It has been shown that xenotransplantation of porcine hUC-MSCs into rat brain did not trigger any immune response after 2-6 wks of follow-up (40). If their nonimmunogenic properties can be confirmed, hUC-MSC cells may potentially serve as a good source of stem cells for the repair of damaged tissues in humans (2, 8, 14, 42). At such circumstance, the ideal type of sera is of importance. Also, any difference between human male and female fetuses cord blood serum could be of importance because it was shown that some materials like leptin concentration are different in male and female fetuses cord blood serum (41). 

In this study, we used 10% hUCS in all experiments. In other studies, depending on the type of cells and experimental conditions, various concentrations of serum supplement have been used. When the autologus serum and FBS are used, the serum concentration and the cell passage number are important factors. For example, at lower cell passages and higher human serum concentrations, the human serum is more effective for adipose cell proliferation (33). Most often, a serum concentration of 5-20% has been suggested (10). Whether a 10% hUCS is the ideal concentration or not, cannot be withdrawn from this study and needs further investigations. In addition, further studies are recommended to understand long-term changes in hUC-MSCs culture in the presence of hUCS and FBS.

## Conclusion

We may conclude that serum-free media could hardly support cell proliferation, while hUCS can be a good substitute for FBS in MSC culture systems. It is more compatible with human cell culture, less expensive and appropriate for stem cell proliferation especially in cell transplantation procedures. 

## Note

***This article extracted from M.D. thesis. (Azadeh Esmaeli)***
